# Do Mothers with Lower Socioeconomic Status Contribute to the Rate of All-Cause Child Mortality in Kazakhstan?

**DOI:** 10.1155/2018/3629109

**Published:** 2018-02-14

**Authors:** Fei Yu, Ziqi Yan, Run Pu, Shangfeng Tang, Bishwajit Ghose, Rui Huang

**Affiliations:** ^1^Peking University First Hospital, Beijing, China; ^2^School of Business and Economics, Zhongnan University of Economics and Law, Wuhan, Hubei, China; ^3^China National Center for Biotechnology Development, Beijing, China; ^4^School of Medicine and Health Management, Tongji Medical College, Huazhong University of Science & Technology, Wuhan, Hubei, China; ^5^School of Pharmacy, Tongji Medical College, Huazhong University of Science & Technology, Wuhan, Hubei, China

## Abstract

**Background:**

This study aimed to explore whether or not mothers with higher educational and wealth status report lower rate of child mortality compared to those with less advantageous socioeconomic situation.

**Methods:**

Data used were cross-sectional and collected from Multiple Indicator Cluster Survey in Kazakhstan conducted in 2015. Subjects experiencing childbirth were 9278 women aging between 15 and 49 years. The associations between maternal education and household wealth status with child mortality were examined by multivariate analytical methods.

**Results:**

The overall prevalence of child mortality was 6.7%, with noticeable variations across the different regions. Compared with women who had the highest educational status, those with upper and lower secondary were 1.47 and 1.89 times more likely to experience child death. Women in the lowest and second lowest wealth quintile had 2.74 and 2.68 times higher odds of experiencing child death compared with those in the richest wealth status households.

**Conclusions:**

Policy makers pay special attention to improving socioeconomic status of the mothers in an effort to reduce child mortality in the country. Women living in the disadvantaged regions with poor access to quality health care services should be regarded as a top priority.

## 1. Background

Health and well-being of an individual are determined to a great extent by the proximal and distal factors ranging from genetic susceptibility, dietary, and nutritional habit to living environment, income, and awareness about personal health [1–6]. The concept of social determinants of health (SDOH) has been used as a reliable tool to investigate the underlying factors that affect heath and disease outcomes which are essentially a result of social rather than health system performance [7–9]. The degree to which a community or an individual is capable of understanding and practicing the fundamental concepts of health related behaviour (tobacco smoking, alcohol drinking, and physical activity) is very much dependent on the knowledge base an individual is being exposed to, which in turn is dependent on having access to information and resources that act as enabling factors of living a healthy life [10–13]. The evidence on the association between socioeconomic and cultural factors on population health is practically universal as poor households in both developed and developing countries share higher risk overall morbidity and mortality compared to their richer counterparts [14–16]. However, the concept of the intergenerational effects of socioeconomic status on health and nutritional status is still in its infancy and deserves exploration from various sociodemographic and geographic aspects.

Mothers with higher educational experience and better economic autonomy are likely to enjoy the resources required to maintain a healthy lifestyle and have better access to health services [17, 18], which are considered to have direct influences on pregnancy outcomes and health of the new-born. From population health perspective, maternal socioeconomic status is thus a matter of particular importance as it serves as a strong determinant of child health. Moreover, infant/child mortality rates are regarded as a key demographic indicator that reflect the overall situation of a healthcare system, national development, and well-being of a population [19, 20]. Given this, it becomes no surprise that infant mortality rate was given a special emphasis in the millennium development goals which were adopted as a comprehensive approach to improving global public health situation in 1990 [21]. MDG 4 was dedicated exclusively to reducing under-five mortality globally by two-thirds between 1990 and 2015.

Since the expiration of the deadline of the MDGs in 2015, numerous studies have been published assessing the progress towards the maternal and child mortality related goals regionally and globally. Literature review of these findings reveal that the success of MDG 4 was not even across countries. While some countries have managed to reduce child mortality to the target level, progress in some others was minimal, especially those in sub-Saharan region. In the context of Asia, Kazakhstan is one of the few countries in the central Asian region which was on track to achieve the child health related MDGs. According to UN estimates, under-five mortality rate in Kazakhstan has decreased by about two-thirds during 1990 to 2012 (from 54.1 deaths/1,000 live births to 18.7) [22]. The success story of Kazakhstan is certainly a valuable instance for its less advanced counterparts in Asia. However, in order to have any workable insights, it is essential to generate evidences from population based data from countries that have reached the milestone. In this study, we therefore aim to analyze the more recent data on maternal socioeconomic status and child mortality in Kazakhstan. The data were obtained from the Multiple Indicator Cluster Survey that was conducted in the country in 2015 and contained information on maternal socioeconomic factors such as educational achievement and household wealth status. The survey was explained further in the methods section.

## 2. Methods

### 2.1. MICS Survey

Multiple Indicator Cluster Surveys (MICS) is an international program launched by UNICEF in 1995. The main vision of this program is to assist countries addressing data gaps for monitoring the progress maternal and child health in the developing countries. The program is currently operating in about 90 developing countries and provide high quality nationally representative data to aid evidenced-based policy making and assess the performance of health-related programs in these countries. MICS has cooperation with Demographic and Health Surveys (DHS) which is also an international health survey program. Supported by the technical and financial assistance from UNICEF and its partners, the national government institutions in the individual countries play the role of carrying out the surveys.

The datasets are internationally comparable and thus serve as an important tool to evaluate and monitoring country progress towards national goals and international commitments [22]. Kazakhstan Multiple Indicator Cluster Survey (MICS) 2015 was the third of this kind to be conducted in Kazakhstan since the year 2005. The survey was conducted by the Statistics Committee of the Ministry of National Economy with the technical and financial support by UNICEF and the UN Population Fund (UNFPA).

Sample areas included urban and rural areas across 16 administrative districts in the country: Akmola, Aktobe, Almaty oblast, Atyrau, West Kazakhstan, Zhambyl, Karaganda, Kostanai, Kyzylorda, Mangistau, South Kazakhstan, Pavlodar, North Kazakhstan and East Kazakhstan regions, Astana, and Almaty. Sampling procedure involved two stages by taking urban and rural areas as the main sampling strata. In total 16 urban and 14 rural areas were included to produce 30 strata for the survey. The first stage of the sampling involved selection of enumeration areas (EAs) with probability proportional to size for each of the 30 strata. At the second stage, individual households were selected systematically within each EAs (20 households per EA), to generate a total sample size of 16,800 households. The questionnaires used in the survey were based on the MICS 5 model questionnaires in English and Russian versions. The questionnaires in the Kazakh and Russian languages were pretested selected areas and were customized for the survey and translated into the Kazakh language. Data collection lasted from early September till late November of 2015. In the households selected for interview, 12,910 women (aged 15–49 years) were identified, and among them 12,670 were successfully interviewed (response rate = 98.1%). More details regarding MICS surveys are available on the final reports [22].

### 2.2. Selection of Variables

The dependent variable was experience of child mortality reported by the participants. The answers were numerical that ranged from zero to 4 (maximum number of death reported). Given the very little frequency of experiencing more than one death, the categories were collapsed into two: yes (child death of any frequency) and no (no child death).

As the main independent variables were socioeconomic status which is usually a complex construct that encompasses income, social benefits, education, and components, for this study two variables were used as proxy measures of this construct: (1) educational attainment (categorized as lower secondary/upper secondary/technical, and professional/higher), and (2) wealth status (categorized as poorest (first)/second/middle/fourth/richest (fifth)).

MICS surveys do not use direct income to measure economic status. Instead it uses a composite measure of household wealth status by taking into calculation household possession of durable goods (TV, Refrigerator, construction material, car, etc.) and then ranks the households according to the score based on the total amount of resources owned by the households [17].

To adjust the analysis for potential confounders, the following were included based on their relevance to child mortality from the current literature: age (15–19/20–24/25–29/30–34/35–39/40–44/45–49), residency (urban/rural), ethnicity (Kazakh/Russian/others), frequency of reading newspaper/magazine (almost every day/at least once a week/less than once a week/not at all), frequency of listening to radio (almost every day/at least once a week/less than once a week/not at all), and ever had aborted pregnancy (yes/no).

### 2.3. Data Analysis

All analyses were performed with STATA®14 for Mac. Selection criterion was having the experience of at least one childbirth. The baseline characteristics of the participants, including the prevalence of child mortality were presented by frequencies and percentages. The association between dependent and explanatory variables was estimated by Chi-square tests. The explanatory variables, which showed significant associations with child mortality, were selected for multivariable regression analysis. Generalized estimating equations method with robust estimator was used to account for the sampling structure. The results of regression were reported as unadjusted odds ratios (Model 1) and then adjusted for the covariates (model 2) along with their 95% confidence interval. *p*-value of <.05 (two-tailed) was considered statistically significant.

## 3. Results

### 3.1. Descriptive Statistics

Table 1 presents the demographic, socioeconomic, media use information of the participants. It shows that most of the women were 40–44 years of age (18.9%) and about three-fifth were of urban origin (59.3%). About a quarter (23.3%) had upper secondary level education and more than one-third technical and professional level qualification (34.9%). Regarding wealth status, less than a quarter (23%) were living in the households with the highest wealth quintile and less than a fifth were in the poorest wealth quintile (18.2%). About two-thirds were of Kazakh ethnicity (65.5%) and 22.5% were of Russian origin. Regarding media use status, 12.3% of women reported reading newspaper or magazine almost every day and 41.1% at least once a week. Regarding radio use on the other hand, 13% women reported listening to radio on daily basis and 65.7% never listening to it.

Table 2 shows the prevalence of child mortality across sixteen different regions in the country. Overall prevalence of child mortality was 6.7%. According to the results, South Kazakhstan, Kyzylorda, and Zhambyl had the highest rates of child mortality in the country.

### 3.2. Chi-Square Tests of Association

Table 3 shows the results of chi-square tests of association between experiencing child death with the explanatory variables. It is clear that the child death was more likely to occur among women ageing 45–49 years, living in the rural areas, having upper-secondary level education, living in the households with poorer wealth status, being of Kazakh ethnicity, not listening to radio, and having history of aborted pregnancy. These variables were subsequently selected for regression analysis in the final step.

### 3.3. Results of Multivariable Regression Analysis

Table 4 shows the results of multivariable regression analysis on the association between child mortality and maternal socioeconomic status. Both in the univariate (Model 1) and in the adjusted model (Model 2), a linear trend in the association between educational level and child death was observed. In the adjusted model, the strength of association (odds ratio) reduced slightly for upper secondary and technical/professional category but increased for that of the lower secondary. In comparison to women who had the highest educational status, those on the lower, such as upper and lower secondary, were 1.47 [OR = 1.471, 95% CI = 1.156–1.871] and 1.89 [OR = 1.896, 95% CI = 1.337–2.688] times more likely to experience child death.

Similar situation was observed for wealth status as well since women in the households with lower wealth quintile had significantly higher odds of reporting child death. Compared to those in the highest (richest) wealth status households, those in the lowest and second lowest had 2.74 and 2.68 times higher odds of experiencing child death.

## 4. Discussion

Child mortality is used as a measure to evaluate the overall socioeconomic progress of a nation. Hence, from both public health and national development perspective it is a necessary imperative to take policy actions to address the causes of child mortality. To facilitate this, undertaking population based studies with an aim to measure the prevalence rates and understand the root causes is particularly important. Based on the Multiple Indicator Cluster Survey data, in the present study, we aimed to explore the association between maternal socioeconomic status and child mortality in Kazakhstan. Our findings suggest that the prevalence was comparatively lower than previous estimates in 2012. The progress might be attributable to continued effort to ensure better access to quality care and wider provision of maternal healthcare services. However, important regional disparities were observed in the distribution of child mortality rates across the country. South Kazakhstan had the highest rates of child mortality (12%) compared to 2.2% in Astana, the capital city. This variation is hard to explain in light of the present analysis; however, it is assumable that, besides better income and living standards, healthcare and living facilities are also most advanced in the capital city/major cities. Women living in the rural areas were more likely than their urban counterparts to report ever experiencing child death. Intuitively, women in the rural areas are less likely to be able to get emergency care and access to special care needs. This phenomenon is not unique to Kazakhstan as even in the most developed countries residents located in the remote areas are less likely to have access to care compared to those living in the city areas [23–25]. Reaching the geographically backward regions in vast countries like Kazakhstan is certainly a challenging task. This needs to be addressed by making policies to reduce regional disparities in access to healthcare services especially for vulnerable groups such as expectant mothers and children.

As expected, the prevalence of child mortality was also higher among women with lower educational level and poorer household wealth status. The relationship between socioeconomic position and health outcome is not a straightforward one as people from different socioeconomic class might be exposed to different types of health risks. Education seems to have wide ranging impact on health outcomes by influencing the level of awareness and maintain good nutrition and hygiene and self-motivation to avoid risky behaviours [26, 27]. Moreover, educated women are more likely to use social media and communicate with peers about the information being shared than those with inadequate educational experience [28, 29]. Recent studies have shown the benefits of utilization of social media on acquiring health and disease related information and health awareness [30–34]. Women who are able to access social and health media are also more likely to seek information regarding the health of their children and are more likely to recognize early symptoms and seek professional care. This altogether can reduce the exposure to disease conditions, rate of morbidity, and mortality of children [35].

Besides health awareness, educated mothers are more likely to be self-independent [36], and have better source of income and are more likely to be able to afford medical care for their children. Experience from developing countries suggests that educated mothers are more likely to be aware of and utilize immunization services which are vital to reduce the burden of child mortality in resource poor settings [37]. Infectious diseases (diarrhea) represent a major group of disease and mortality burden in the developing countries, which could have been avoided by practicing proper hygiene methods at home. Mothers who are unaware of these knowledge are more likely to encounter hygiene related illnesses among children.

From the discussions above, it is clear that maternal education and economic status have significant association with child death in Kazakhstan. It is important to mention that the present study only includes mortality cases reported by mothers and not any disease status. It is possible that children living in the poorest households are exposed to frequent episodes of infectious diseases and poor nutritional status. In a Bolivian study (Bolivia Demographic and Health Survey), socioeconomic factors were found to be the most important link between maternal education and nutritional status of children [38]. Similar findings were replicated in studies conducted on countries in Sub-Saharan Africa such as Ghana [39] and Kenya [40]. Based on these discussions and the findings of the present analysis, it is worthy of recommending that the policy makers in the country place special focus on addressing the socioeconomic inequalities among women which can eventually pay off in the aggregate in terms of reduced child mortality and better nutritional status of mothers and their children. Providing universal education and healthcare access to mothers can be very productive in this respect. Moreover, causes of regional disparities need to be explored and addressed to make sure geographical barrier not interfere with healthcare seeking among mothers and their children in the remote areas.

## 5. Strengths and Limitations

This is the first study to estimate the prevalence of child mortality in association with maternal socioeconomic status in Kazakhstan. A particular strength of this study was that the sample size was relatively large and nationally representative. Data were collected from the most recent survey. However, there are several limitations that can potentially impact the outcomes of the study. Firstly, the age of the children was not recorded. Interviewers asked whether the participant had any child who died. So the causes of death were also not possible to consider in this analysis. Moreover, as the data were cross-sectional, there was no indication of causality or directionality of the association between maternal socioeconomic status and child mortality.

## 6. Conclusions

The present study reports the prevalence of all-cause child mortality in Kazakhstan and its association with maternal socioeconomic status. The findings indicate that lower educational and household wealth status were associated with higher odds of child mortality. In other words, children born to mothers who were better educated and lived in better economic condition had higher survival rates. However, it was not clear from the data to what extent the beneficial effect resulted from maternal socioeconomic status. As the study could not adjust the analysis for disease specific risk factors of the children and the health status of mothers, the association remains subject to further exploration. Nonetheless, the findings provide important insight for health policy makers in the country. Based on the analysis it is suggestible that addressing socioeconomic inequality could lead to better child survival rate in the country. Future studies should focus on assessing the relationship by including more diverse range of maternal and child health related variables.

## Figures and Tables

**Table 1:** Basic demographic and socioeconomic characteristics of the sample population, MICS 2015.

	*N* = 9.278	%
Age		
15–19	55	0.6
20–24	758	8.2
25–29	1629	17.6
30–34	1748	18.8
35–39	1721	18.5
40–44	1750	18.9
45–49	1617	17.4
Residency		
Urban	5501	59.3
Rural	3777	40.7
Educational attainment		
Lower secondary	518	5.6
Upper secondary	2148	23.2
Technical and professional	3237	34.9
Higher	3375	36.4
Wealth status		
Poorest	1693	18.2
Second	1570	16.9
Middle	1910	20.6
Fourth	1968	21.2
Richest	2137	23.0
Ethnicity		
Kazakh	6077	65.5
Russian	2084	22.5
Other ethnic groups	1117	12.0
Frequency of reading newspaper/magazine		
Almost every day	1145	12.3
At least once a week	3841	41.4
Less than once a week	2211	23.8
Not at all	2081	22.4
Frequency of listening to radio		
Almost every day	1205	13.0
At least once a week	1092	11.8
Less than once a week	888	9.6
Not at all	6093	65.7
Ever had aborted pregnancy		
Yes	2598	28.0
No	6679	72.0

**Table 2:** Regional estimates of child mortality in Kazakhstan.

		Children dead		Total
		No (93.3%)	Yes (6.7%)	
Region	Akmola	6.6	7.5	6.7
	Aktobe	5.8	2.6	5.5
	Almaty oblast	5.8	4.5	5.7
	Atyrau	6.0	5.6	6.0
	West Kazakhstan	6.1	2.9	5.9
	Zhambyl	6.4	10.4	6.6
	Karaganda	5.8	4.6	5.7
	Kostanai	7.3	6.4	7.2
	Kyzylorda	6.6	10.6	6.9
	Mangistau	6.5	7.9	6.6
	South Kasakhstan	7.0	12.0	7.3
	Pavlodar	6.3	4.5	6.1
	North Kazakhstan	6.0	6.6	6.0
	East Kazakhstan	5.3	6.3	5.4
	Astana City	6.2	2.2	5.9
	Almaty City	6.6	5.4	6.5

Total		100%	100%	100%

**Table 3:** Bivariate association between child mortality and maternal socioeconomic status.

Variables	Experienced child death		*p*-value
	No	Yes	
Age			.0001
15–19	0.6%	1.1	
20–24	8.6%	1.6%	
25–29	18.1%	8.5%	
30–34	19.2%	13.3%	
35–39	18.6%	18.4%	
40–44	18.4%	25.5%	
45–49	16.4%	31.6%	
Residency			
Urban	60.8%	38.1%	.0001
Rural	39.2%	61.9%	
Educational attainment			
Lower secondary	5.3%	9.0%	.0001
Upper secondary	22.3%	35.1%	
Technical and professional	35.0%	33.3%	
Higher	37.4%	22.6%	
Wealth status			
Poorest	17.4%	30.4%	.0001
Second	16.3%	25.5%	
Middle	20.6%	20.7%	
Fourth	21.7%	13.9%	
Richest	24.0%	9.5%	
Ethnicity			
Kazakh	65.1%	71.6%	.001
Russian	23.0%	15.5%	
Other ethnic groups	12.0%	12.8%	
Frequency of reading newspaper/magazine			
Almost every day	12.3%	12.8%	.978
At least once a week	41.4%	41.5%	
Less than once a week	23.8%	23.7%	
Not at all	22.5%	22.0%	
Frequency of listening to radio			.002
Almost every day	13.1%	11.4%	
At least once a week	12.0%	9.1%	
Less than once a week	9.8%	6.9%	
Not at all	65.2%	72.6%	
Ever had aborted pregnancy			
Yes	27.5%	34.6%	.001
No	72.5%	65.4%	

**Table 4:** Association between educational and wealth status of women and child death in Kazakhstan.

	Model 1	Model 2
	Odds ratio (95% CI)	Odds ratio (95% CI)
Educational attainment		
Lower secondary	1.789 (1.276, 2.507)	1.896 (1.337, 2.688)
Upper secondary	1.761 (1.395, 2.223)	1.471 (1.156, 1.871)
Technical and professional	1.312 (1.049, 1.642)	1.146 (0.911, 1.442)
Higher	Ref	Ref
Wealth status		
Poorest	3.490 (2.549, 4.778)	2.740 (1.898, 3.954)
Second	3.344 (2.443, 4.577)	2.683 (1.867, 3.855)
Middle	2.272 (1.653, 3.124)	2.083 (1.488, 2.915)
Fourth	1.558 (1.112, 2.183)	1.538 (1.095, 2.162)
Richest	Ref	Ref

## Data Access

The datasets are available in the public domain of the MICS website in anonymized form.
